# Supplementary material for: Antennal Transcriptome Analysis and Identification of Candidate Chemosensory Genes of the Harlequin Ladybird Beetle, Harmonia axyridis (Pallas) (Coleoptera: Coccinellidae)
Source: Insects. 2021 Mar 2;12(3):209. doi: 10.3390/insects12030209 (PMC8002065; doi:10.3390/insects12030209)
Supplement: Supplementary file 1 [file insects-12-00209-s001.zip › Supplementary_files_/Table_S10_SNMPs_.docx]

| Table S10: Best similarity for sensory neuron membrane proteins (SNMPs) of *Harmonia axyridis* (Haxy) | | | | | | | | | |
| --- | --- | --- | --- | --- | --- | --- | --- | --- | --- |
| Name | ID | length (aa) | ORF status | TMHMM | Bit score | E-value | % identity | Accession number | Best Blast hit of the longest isoforms |
| HaxySNMP1.1 | TRINITY_DN24414_c1_g1::TRINITY_DN24414_c1_g1_i2 | 525 | 3prime_partial | 2 | 688 | 0 | 62.500 | XP_001816436 | PREDICTED: sensory neuron membrane protein 1 [Tribolium castaneum] |
| HaxySNMP1.2 | TRINITY_DN24697_c2_g1::TRINITY_DN24697_c2_g1_i1 | 554 | 5prime_partial | 2 | 533 | 0 | 51.329 | ALR72543 | sensory neuron membrane protein SNMP1b [Colaphellus bowringi] |
| HaxySNMP2.1 | TRINITY_DN5381_c0_g1::TRINITY_DN5381_c0_g1_i1 | 205 | 5prime_partial | 1 | 270 | 1.54e-85 | 63.636 | XP_008198962 | PREDICTED: sensory neuron membrane protein 2 [Tribolium castaneum] |
| HaxySNMP2.2 | TRINITY_DN5381_c0_g2::TRINITY_DN5381_c0_g2_i1 | 207 | 3prime_partial | 1 | 216 | 9.94e-65 | 49.029 | XP_008198962 | PREDICTED: sensory neuron membrane protein 2 [Tribolium castaneum] |
