# Supplementary figures and images for: Antennal Transcriptome Analysis and Identification of Candidate Chemosensory Genes of the Harlequin Ladybird Beetle, Harmonia axyridis (Pallas) (Coleoptera: Coccinellidae)
Source: Insects. 2021 Mar 2;12(3):209. doi: 10.3390/insects12030209 (PMC8002065; doi:10.3390/insects12030209)

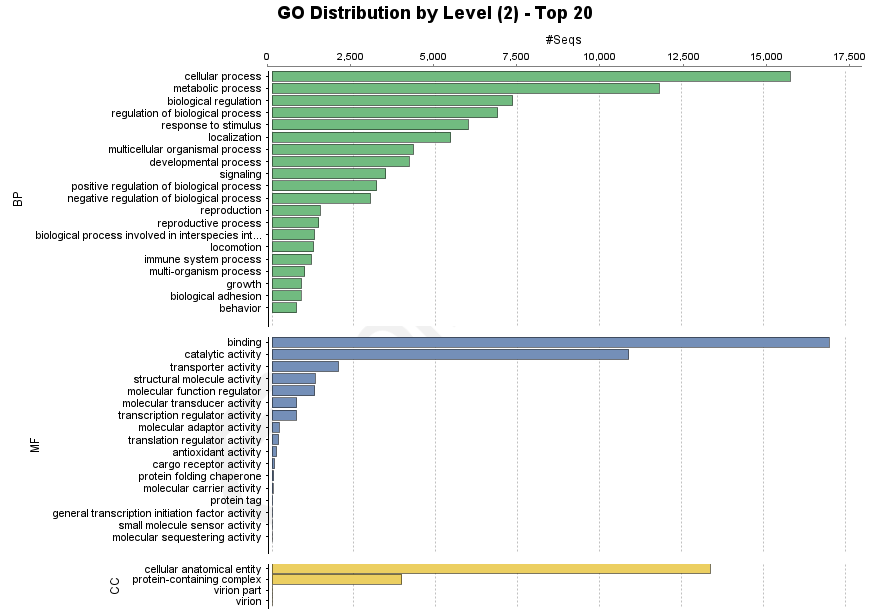

Supplement: Supplementary file 1 [file insects-12-00209-s001.zip › Supplementary_files_/Figure_S1.png]

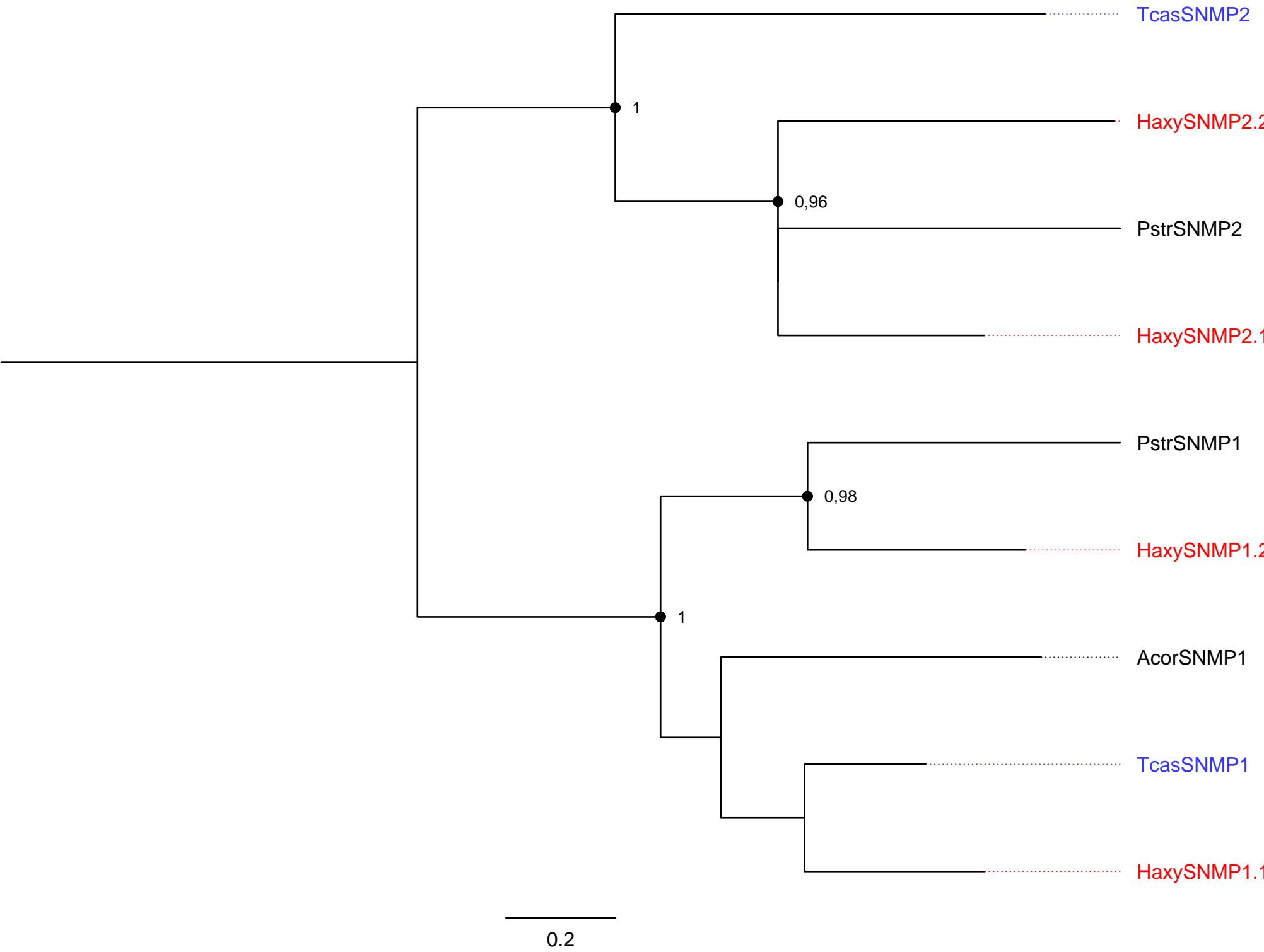

Supplement: Supplementary file 1 [file insects-12-00209-s001.zip › Supplementary_files_/Figure_S2.pdf]
